# Forecasting COVID-19 confirmed cases, deaths and recoveries: Revisiting established time series modeling through novel applications for the USA and Italy

**DOI:** 10.1371/journal.pone.0244173

**Published:** 2021-01-07

**Authors:** Emrah Gecili, Assem Ziady, Rhonda D. Szczesniak

**Affiliations:** 1 Division of Biostatistics & Epidemiology, Cincinnati Children’s Hospital Medical Center, Cincinnati, OH, United States of America; 2 Department of Pediatrics, Cincinnati Children’s Hospital Medical Center, Cincinnati, OH, United States of America; 3 Department of Pediatrics, University of Cincinnati, Cincinnati, OH, United States of America; Faculty of Science, Ain Shams University (ASU), EGYPT

## Abstract

The novel coronavirus (COVID-19) is an emergent disease that initially had no historical data to guide scientists on predicting/ forecasting its global or national impact over time. The ability to predict the progress of this pandemic has been crucial for decision making aimed at fighting this pandemic and controlling its spread. In this work we considered four different statistical/time series models that are readily available from the ‘forecast’ package in R. We performed novel applications with these models, forecasting the number of infected cases (confirmed cases and similarly the number of deaths and recovery) along with the corresponding 90% prediction interval to estimate uncertainty around pointwise forecasts. Since the future may not repeat the past for this pandemic, no prediction model is certain. However, any prediction tool with acceptable prediction performance (or prediction error) could still be very useful for public-health planning to handle spread of the pandemic, and could policy decision-making and facilitate transition to normality. These four models were applied to publicly available data of the COVID-19 pandemic for both the USA and Italy. We observed that all models reasonably predicted the future numbers of confirmed cases, deaths, and recoveries of COVID-19. However, for the majority of the analyses, the time series model with autoregressive integrated moving average (ARIMA) and cubic smoothing spline models both had smaller prediction errors and narrower prediction intervals, compared to the Holt and Trigonometric Exponential smoothing state space model with Box-Cox transformation (TBATS) models. Therefore, the former two models were preferable to the latter models. Given similarities in performance of the models in the USA and Italy, the corresponding prediction tools can be applied to other countries grappling with the COVID-19 pandemic, and to any pandemics that can occur in future.

## Introduction

Cases of severe respiratory illness began to be reported across the city of Wuhan in China in December 2019. These were caused by a new type of coronavirus, SARS-CoV2, alternatively known as COVID-19 [1]. The number of COVID-19 cases quickly escalated in mid-January, and the virus soon spread beyond China's borders. As of this study, it has reached 188 countries worldwide [2]. Despite broadening COVID-19 impacts, there remain key public health questions. How many people will be infected? How does the situation change day by day? Can we predict/forecast the future numbers of people infected with COVID-19 by using daily updated data on the pandemic’s trajectory? These questions could be answered by forecasting the possible futures of this pandemic through statistical/time series models. However, widely used time series models and tools do not necessarily have high forecasting/prediction accuracy, especially for medical studies [3]. As the COVID-19 pandemic evolves and additional data are amassed [4], new insights emerge on the reasonableness of the prediction models developed and tested; however, predicting its future requires transparent reporting and multiple model assessments [3]. Despite these ongoing challenges, forecasting is still critical to better understand the current situation and progression of COVID-19, to prepare for the future of this ongoing pandemic. Using appropriate models and making accurate projections consistently may enhance public health decision-making; for example, intervention-induced changes in the spread of disease [5]. In this paper, we provide statistical forecasts for the confirmed cases of COVID-19 using four different, highly cited time series models, which we subsequently introduce, and compare their performance to analyze the trajectory of cases.

In this work, we propose examining the utility of these models for forecasting the number of new COVID-19 cases in select countries based on the daily reported data. By empirically comparing multiple models in terms of their forecasting accuracy, we intend to suggest an appropriate model, which can be readily used by society, organizations, or governments to assess near futures of this outbreak. We acknowledge that this is a difficult forecasting problem, since this pandemic continues and there are many factors that we cannot presently control. Our prediction accuracy will improve with time and more data. An appealing part of these models that we considered is that they are already implemented in an accessible R package and can be applied readily by analysts. Fitting each model only takes several seconds, and the models are conducive to daily updating as newly available data are accrued. Daily updating of these models is suggested, because the accuracy of the forecasting depends on the available data used for model development, producing predictions, and generating prediction intervals (PIs) [4].

## Materials and methods

We considered four time series models for our case study. The first model was the Holt model [6, 7], which employs double exponential smoothing; two smoothing components adjust for level and trend in the data. The second model was the Autoregressive Integrated Moving Average (ARIMA) model, which theory dictates is the most general class of models for forecasting a time series. Although the ARIMA model is useful and powerful in time series analysis, it is somewhat difficult to choose appropriate orders for its components. For our purposes aimed at utility, it was necessary to determine the orders automatically; thus, we used the ‘auto.arima’ function from the forecast package in R, which returns the best ARIMA model according to model fit statistics by searching over possible models within the order constraints provided. Third was the TBATS (Trigonometric Exponential smoothing state space model with Box-Cox transformation, ARMA errors, Trend and Seasonal component) model [8], which uses a combination of Fourier terms with an exponential smoothing state space model and a Box-Cox transformation, in a completely automated manner. The last model considered was the cubic smoothing spline model based on a stochastic state space model that allows the use of a likelihood approach for estimating the smoothing parameter, thereby facilitating construction of the PI. It has been shown that this model is a special case of an ARIMA(0, 2, 2) model [9]. Its advantage over the full ARIMA model is that it provides a smooth historical trend as well as a linear forecast function. An overview of the models is provided as S1 Table in S1 File, along with specific equations.

The main outcomes of interest were the daily collected numbers of confirmed cases, deaths, and recoveries. We downloaded the data from the Center for Systems Science and Engineering (CSSE) at Johns Hopkins University (https://github.com/CSSEGISandData/COVID-19 accessed on 04/28/2020), which consists of daily cumulative cases and covered our analysis period from February 22, 2020 until April 29, 2020. The unit of time used in modeling was day. Each outcome, beginning between March and April, exhibited a nearly exponential increase over time for both the USA and Italy (see S1 Fig in S1 File).

The forecasting performance of all these models were evaluated using mean absolute error (MAE) and mean absolute percentage error (MAPE). Additionally, the model fits were evaluated by using AIC (Akaike information criteria), reported in Table 1. We examined forecast durations in a weekly fashion, to assess model performance as extent of data collection increased over calendar time. All the aforementioned models are already implemented and available in the ‘forecast’ package in R, which makes implementation straightforward and possible in freely available software. The computer code that we used for our analyses is provided as S1 File.

**Table 1:** Accuracy metrics of forecasting all three outcomes in the USA and Italy for each model and forecasting period.

	Period I			Period II			Period III			Period IV			Period V				
	Apr 2-Apr 8			Apr 9-Apr 15			Apr 16-Apr 22			Apr 23-Apr 29			Apr 30-May 6				
**Model**	MAE	MAPE	AIC	MAE	MAPE	AIC	MAE	MAPE	AIC	MAE	MAPE	AIC	MAE	MAPE	AIC	Country	Outcome
ARIMA	787	10	692	1021	8.7	829	1160	7.6	959	1250	6.8	1090	1506	6.1	1237	USA	Case
Holt	775	11.3	769	985	9.3	920	1140	8.4	1065	1250	7.6	1212	1506	6.9	1276		
Splines	705	13	653	1011	8.1	835	1231	7.1	1036	1261	6.9	1210	1526	6	1367		
TBATS	743	9.6	611	1048	10.5	769	1305	8.6	925	1354	9.9	1084	1585	8.4	1243		
ARIMA	570	4.9	649	553	4.3	760	548	3.7	872	544	3.4	983	82	7.6	1091	Italy	Case
Holt	565	12	723	543	10.7	848	540	8	974	530	9.4	1100	83	7.8	1225		
Splines	576	7	655	558	6	754	580	5.4	870	543	4.6	980	88	8.9	1095		
TBATS	788	7.8	682	688	7.1	821	630	6	959	562	6.1	1093	95	48.8	1223		
**Model**	MAE	MAPE	AIC	MAE	MAPE	AIC	MAE	MAPE	AIC	MAE	MAPE	AIC	MAE	MAPE	AIC	Country	Outcome
ARIMA	34	17	457	66	14.6	596	80	12.5	698	109	11.6	823	160	9.9	961	USA	Death
Holt	34	20.3	522	60	18	673	79	16.4	794	121	17	951	162	15	1092		
Splines	38	15	511	68	12.3	621	80	11.7	743	136	11.5	891	165	9	1003		
TBATS	47	18.4	525	76	16.2	687	95	15	803	143	13.7	950	172	11.2	1089		
ARIMA	62	7	476	62	6	559	58	5	634	53	4.1	713	56	4.06	796	Italy	Death
Holt	60	25	537	59	7.9	633	58	18	729	58	16.6	824	57	14.6	920		
Splines	63	7.9	474	62	8.2	570	60	7.1	702	76	6.3	789	58	5.5	907		
TBATS	71	8.4	461	84	7.5	573	77	7.2	682	72	6.4	791	65	5.8	897		
**Model**	MAE	MAPE	AIC	MAE	MAPE	AIC	MAE	MAPE	AIC	MAE	MAPE	AIC	MAE	MAPE	AIC	Country	Outcome
ARIMA	133	15.4	582	234	12.6	741	413	10.4	918	534	9.5	1044	905	9.4	1237	USA	Recovery
Holt	130	16	653	238	11.6	827	427	10.6	1020	531	9.6	1162	910	9.9	1371		
Splines	149	19.6	575	319	17.2	698	654	15.3	782	726	12.7	904	1047	9.7	1145		
TBATS	180	14.7	372	607	15.5	522	802	15.7	678	954	65.9	891	1220	17	1005		
ARIMA	106	12	545	101	10.7	635	93	9.5	724	85	8.4	811	82	7.6	898	Italy	Recovery
Holt	106	12.5	609	103	11	713	94.8	9.7	816	86.3	8.6	919	83	7.8	1022		
Splines	114	14.5	549	108	12.5	655	140	14.3	738	90	9.7	889	88	8.9	935		
TBATS	185	16.8	521	135	42.4	670	148	12.1	764	102	54	910	95	48.8	1019		

MAPE (%) and MAE (number of individuals).

## Results

We performed the forecasting analysis for confirmed COVID-19 cases, number of deaths, and number of recoveries for the USA and Italy by using the models discussed above. For both countries, we conducted 7-day- or one-week ahead point forecasts and PIs. We updated our forecasts every 7 days under each of the four models considered. The results of our analyses for both countries are summarized with figures provided below. The accuracy metrics and fit statistics for each forecasting period, outcome, model and country are provided in Table 1.

**Fig 1:**
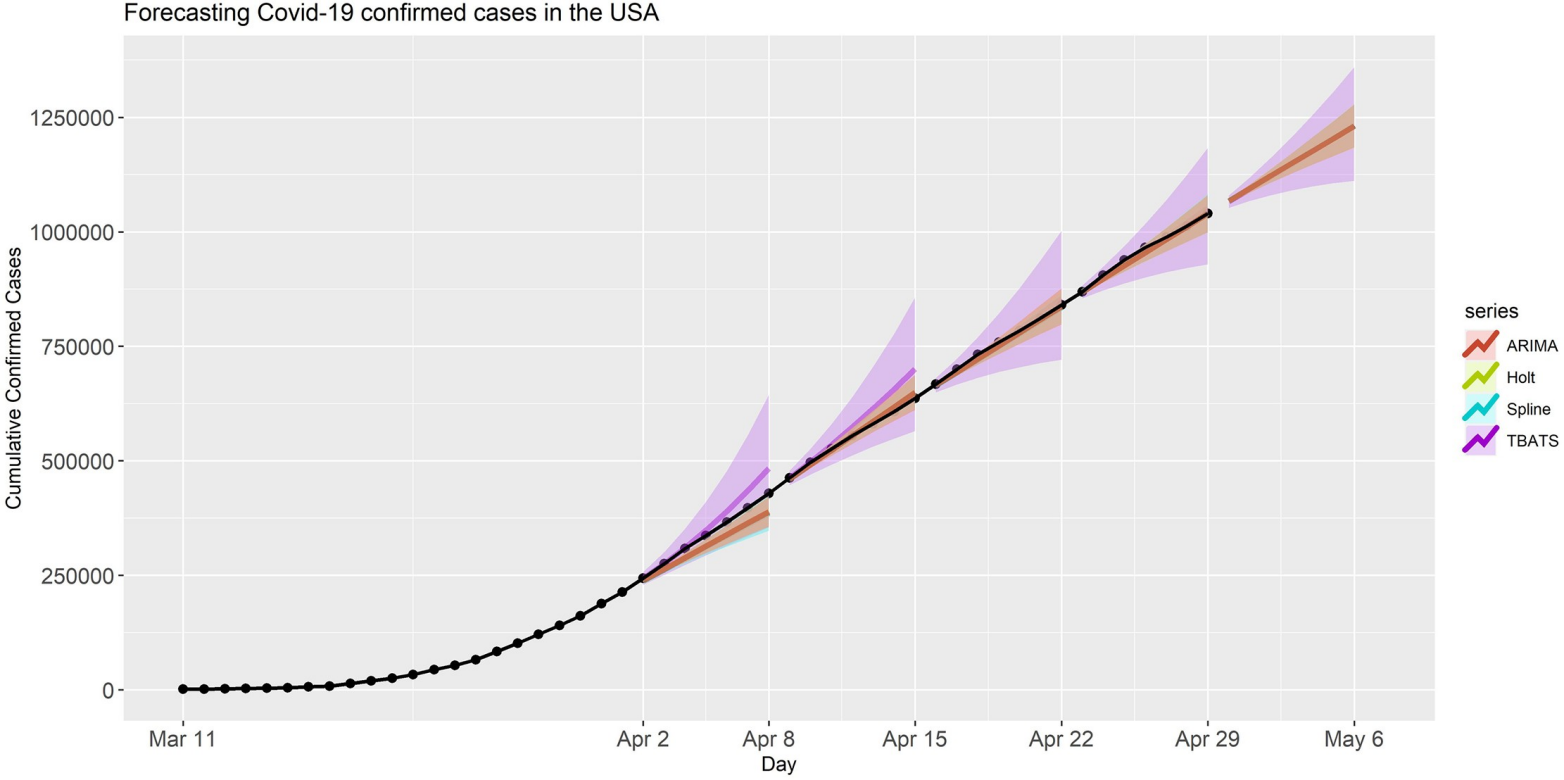
Forecasted confirmed cases in the USA projected under each model and forecasting period. The black dots are the observed confirmed cases. The shaded bands are the 90% prediction intervals for the predictions with various models. The right part of the figure is trimmed to have better visualization wherein we have forecasting periods.

Firstly, we considered applying these models to forecast the number of confirmed cases for the USA. The observed and forecasted confirmed COVID-19 cases in the USA with 90% PIs for each forecasting period and model are presented in Fig 1.

The first forecast period covered April 2- April 8, 2020 where we used the data collected during the previous days. The forecasts and 90% PIs produced at the end of April 1 are presented in Fig 1. For the four models, the mean estimates and accompanying 90% PI for the confirmed cases 7-days ahead, expressed in rounded form in terms of thousands, were 389 (356, 422) for ARIMA, 484 (354, 644) for TBATS, 386 (347, 424) for Spline, and 389 (356, 423) for Holt. The actual number of confirmed cases was 428654 (rounded to 429 in terms of thousands) on April 8, which was captured by only the 90% prediction interval of TBATS model; this model had the lowest MAE and MAPE for this specific forecasting period (see Table 1). In this period predictions were less accurate than expected, possibly due to limited available data till April 1.

For the second forecast period, we incorporated another week of historical data by including the number of cases observed until April 8, 2020. This time the forecast period covered April 9- April 15, 2020. We again used the same models to conduct 7-day ahead predictions. The forecasts and 90% PIs produced at the end of April 7 are presented in Fig 1. The mean estimate (PI) for the confirmed cases 7-days ahead was 649 (611, 687), 701 (565, 857), 650 (616, 683), and 650 (613, 686) for the models ARIMA, TBATS, Spline, and Holt, respectively. The actual number of confirmed cases on April 14 was 636350 (rounded to 636 in terms of thousands), which was included by the 90% prediction intervals of all four models. Among all models, the Spline model had the lowest MAPE 8.1%, followed by the ARIMA model with MAPE 8.7% (see Table 1).

Furthermore, we conducted another set of forecasts and PIs for Period III which includes April 16-April 22, 2020. Similar to the analyses presented above, we used the data up until April 15, 2020. The mean estimate (PI) for 7-days ahead was 837 (797, 876), 854 (721, 1003), 834 (801, 868), and 837 (798, 876) for the models ARIMA, TBATS, Spline, and Holt, respectively. The actual number of confirmed cases on April 22 was 840351 (rounded to 840 in terms of thousands), which was well inside the prediction bands of all four models (see Fig 1). Among these models, the Spline model had the lowest MAPE which is 7.1%, but overall each method had quite small MAPE (see Table 1).

In the forecasting period IV, we considered forecasting for April 23- April 29, 2020 by using the data up until April 22, 2020. The mean estimate (PI) for 7-days ahead was 1040 (999, 1081), 1052 (929, 1183), 1041 (999, 1082), and 1040 (998, 1081) for the models ARIMA, TBATS, Spline, and Holt, respectively. The actual number of confirmed cases on April 29 was 1039909 (rounded to 1040 in terms of thousands) which is aligned with the predicted values by all models and included by their 90% PI. All the models had very low MAPE, slightly differing from each other, especially the Spline and ARIMA models which had MAPE< 7% (see Table 1).

In the final step, we used entire data history up till April 29, 2020, and we attempted forecasting for period V covering April 30 to May 06, 2020. Predicted confirmed cases and corresponding PIs for all models are presented in Fig 1. For this period, the point forecast (PI) for 7-days ahead was 1231 (1184, 1279), 1232 (1111, 1360), 1232 (1185, 1279), and 1231 (1183, 1279) for the models ARIMA, TBATS, Spline, and Holt, respectively. The data on April 29, 2020, were used to provide point forecasts and prediction intervals for the period V (April 30- May 06, 2020). At the time we performed our study, this period had not occurred; thus, we did not have the actual numbers of confirmed cases up to the concluding point of our analyses. The point forecasts and prediction intervals are provided in S2 Table in S1 File. As more data becomes available and is incorporated in the forecasting analysis, the model’s predictive accuracies (e.g., MAPE) improved (see Table 1). For this period, the MAPE values are 6.1%, 8.4%, 6%, and 6.9% for ARIMA, TBATS, Spline, and Holt, respectively.

**Fig 2:**
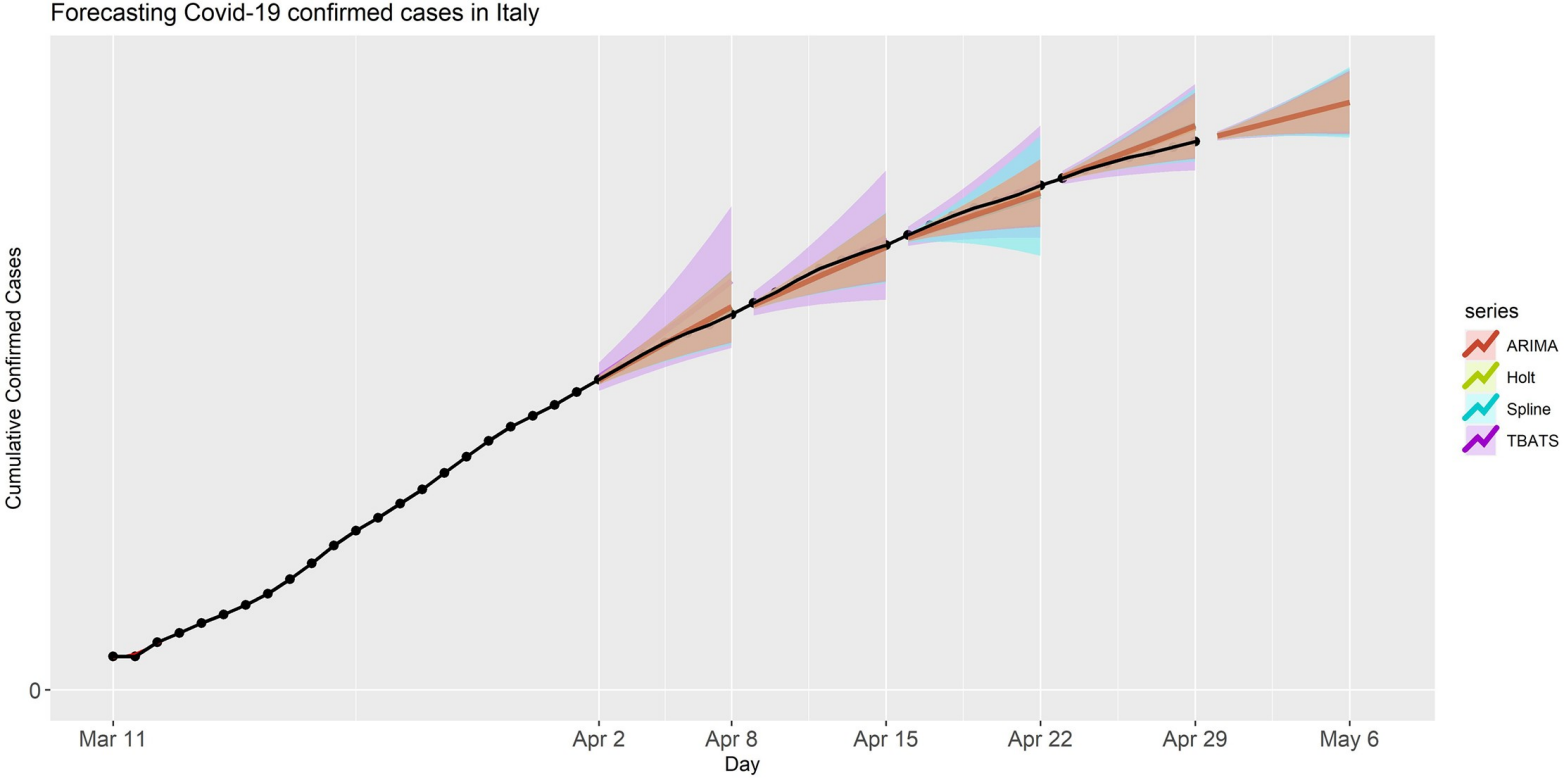
Forecasted confirmed cases in Italy for each model and forecasting period. The black dots are the observed confirmed cases. The shaded band is the 95% prediction interval for corresponding predictions with various models. The right part of the figure is trimmed to have better visualization wherein we have forecasting periods.

Similarly, we applied these models to forecast the confirmed cases for Italy for the same forecasting periods, and the results are presented in Fig 2. Based on our analyses, we concluded that the prediction performance of all these models were similar to what we observed in the USA data application. The values of actual observed confirmed cases were contained inside the prediction bands of all four models for all five forecasting periods. In particular, the models ARIMA, Spline and TBATS had quite small MAPE and good prediction accuracies (see Table 1). The TBATS model had a wider PI for the first four forecasting periods but behaved similar to the PIs of the other three models for period V (see Fig 2). Additionally, the point forecasts and prediction intervals for the period V are provided in S3 Table in S1 File. We observed decreased in the forecasting accuracy metrics of all four models, compared to the previous period in both applications, which is expected as more data becomes available from forecasting periods I to V.

**Fig 3:**
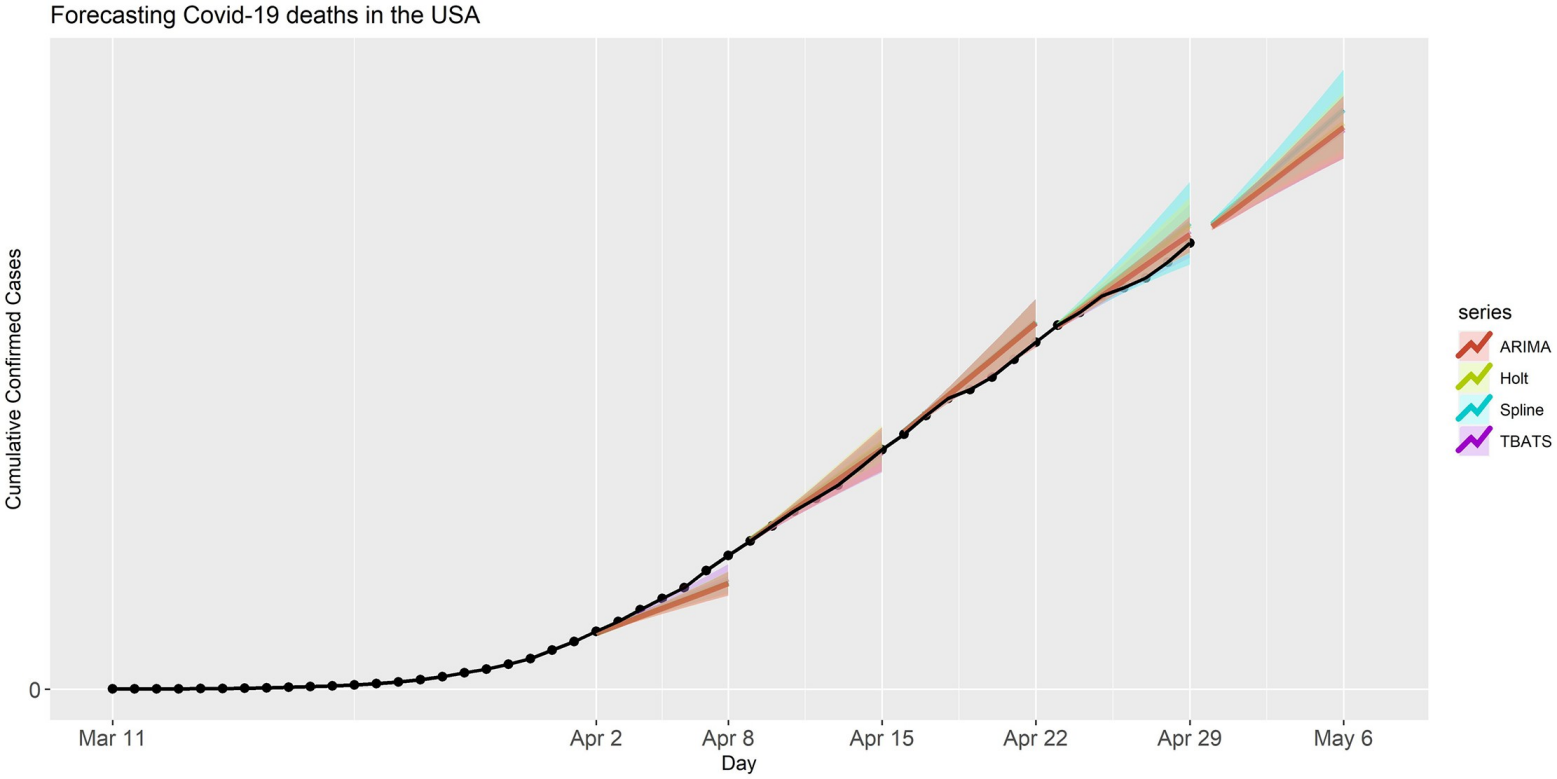
Forecasted deaths in the USA for each model and forecasting period. The black dots are the observed deaths. The shaded band is the 90% prediction intervals for the predictions with various models.

We next applied the same models to forecast the number of deaths due to COVID-19 in the USA for the aforementioned forecasting periods, although these models did not account for all factors that affect mortality of patients infected with COVID-19. The point forecast and PIs that we produced are presented in Fig 3. Again, for this application, the prediction performance of all these models was similar, and the values of actual observed number of deaths were within the PI bands of all four models for at least part of the forecasting periods. The ARIMA and Spline models had smaller prediction errors, compared to the other two models. The forecasting accuracy metrics and AIC values for this application are also provided in Table 1. Overall, the observed number of deaths were included by the PIs from these models except the predictions for forecasting periods I and III (see Fig 3). Additionally, the point forecasts and prediction intervals for the period V (April 30- May 06, 2020) are provided in S4 Table in S1 File. For this set of analyses, the models had higher MAPE than the analyses we produced for the confirmed cases for both the USA and Italy.

**Fig 4:**
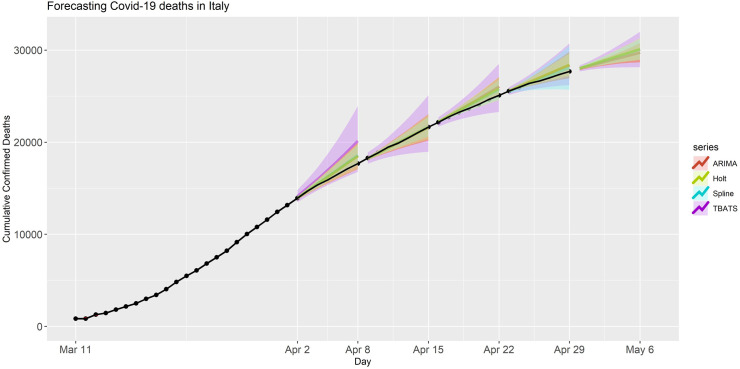
Forecasted deaths in Italy for each model and forecasting period. The black dots are the observed deaths. The shaded band is the 90% prediction intervals for the predictions with various models.

We also conducted forecasting for the number of deaths caused by COVID-19 in Italy by using the same models. Similar to previous analyses, the point forecast and prediction intervals for these analyses were produced and presented in Fig 4. In this application, we observed that the prediction performance of ARIMA, Splines, and TBATS models were better than the performance of the Holt model, which yielded much higher prediction errors compared to others. However, the PIs of all four models included the observed number of deaths for all forecast periods. Similar to previous analyses, the forecast accuracy metrics and model fit statistics of this application are summarized in Table 1. Further, the point forecasts and PIs for period V (April 30- May 06, 2020) are provided in S5 Table in S1 File. For this set of analyses, the models yielded substantially lower MAPE than the analyses that was conducted for the number of deaths in the USA.

**Fig 5:**
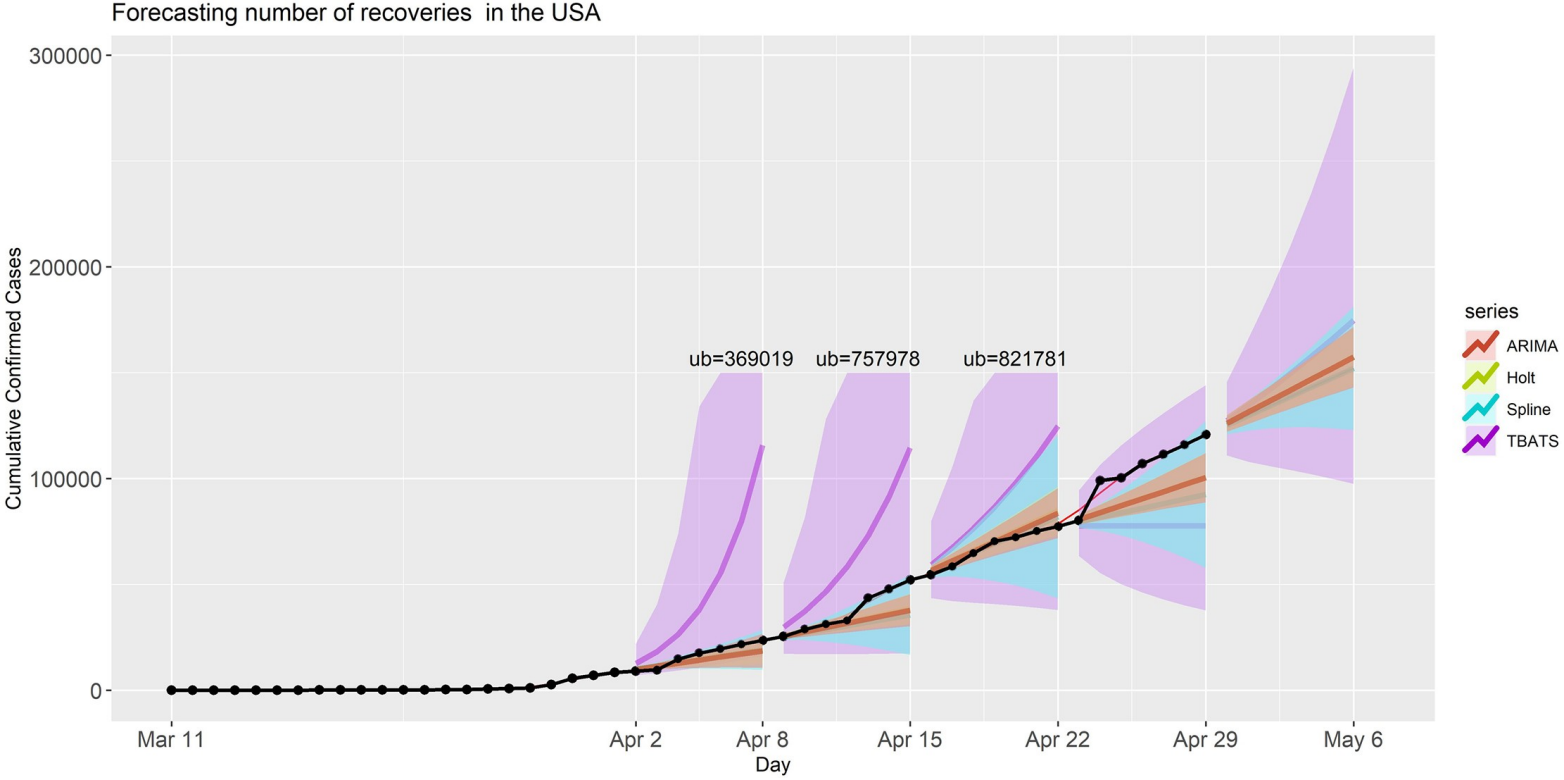
Forecasted recoveries in the USA for each model and forecasting period. Black dots are the observed recoveries. The shaded band is the 90% prediction intervals for the predictions with various models.

We now considered forecasting for the number of recoveries from COVID-19 in the USA by applying the same set of models. We obtained the point forecasts and prediction intervals for different forecast period that are presented in Fig 5. The MAE, MAPE, and AIC values of this analysis are provided in Table 1. The prediction performance of ARIMA, Splines, and Holt models were much better than the performance of the TBATS model, which yielded much higher prediction errors and quite wide PIs, especially for the first three forecasting periods in this application (for these periods the prediction intervals of TBATS model are trimmed to have better visualization in Fig 5). However, the performance of the TBATS model improved as more data became available. The PIs from the other three models did not contain the observed number of recoveries for forecast period IV: likely due to an unexpected jump/change in the trajectory of the number of recoveries in the USA. The forecasting accuracy metrics for this application are summarized in Table 1. The point forecasts and PIs for period V (April 30- May 06, 2020) are presented in S6 Table in S1 File.

**Fig 6:**
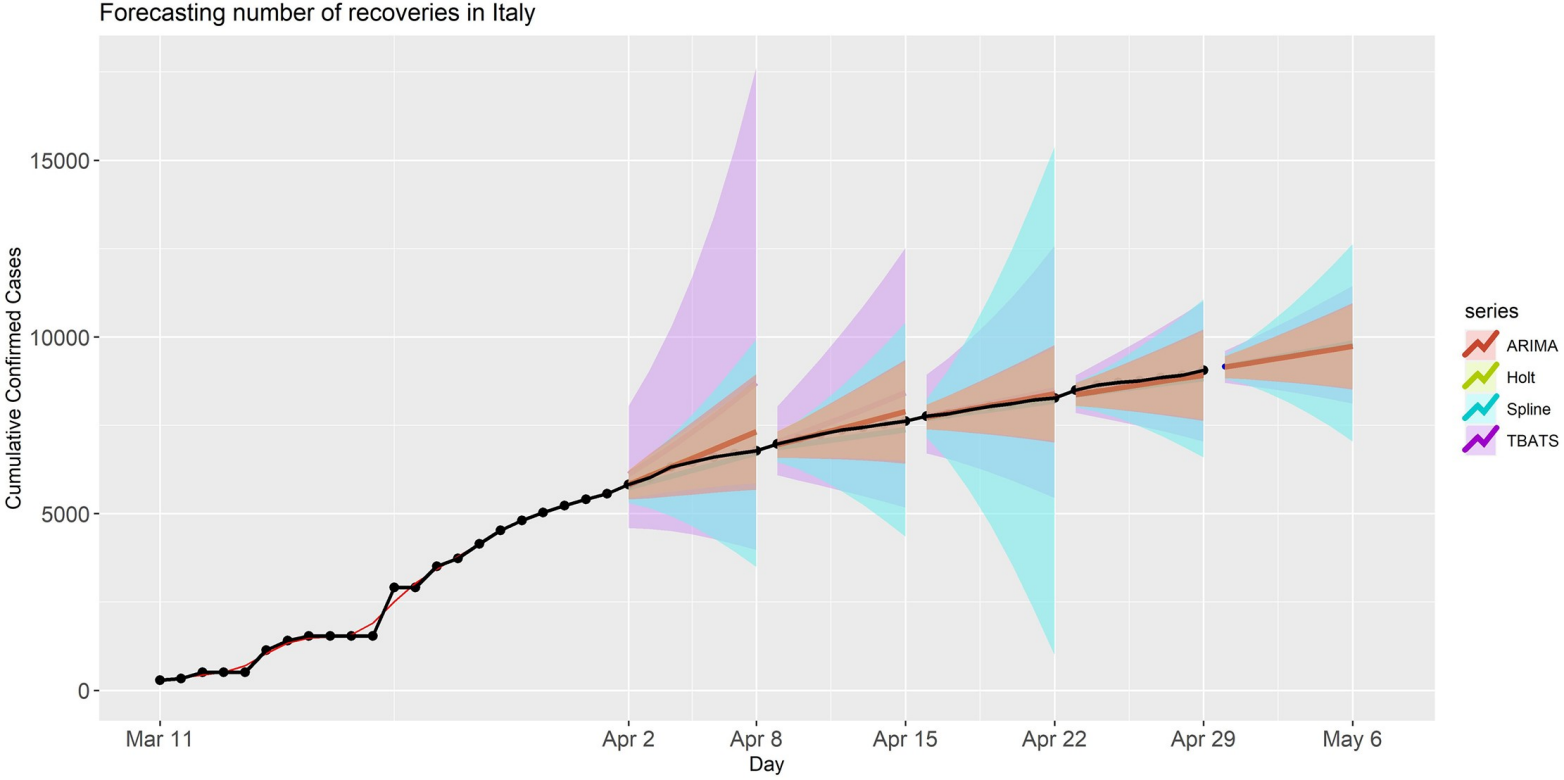
Forecasted recoveries in Italy for each model and forecasting period. The black dots are the observed recoveries. The shaded band is the 90% prediction intervals for the predictions with various models.

Similarly, we then applied the model to generate forecasts for the number of recoveries from COVID-19 in Italy. Point forecasts and PIs for the aforementioned forecast periods are presented in Fig 6. For this application, we found that the prediction performance of ARIMA and Holt models were slightly better than the performance of the Spline and TBATS models. The TBATS model again had poor performance compared to the other models in the analyses for the first two forecasting periods, but its performance improved for the later periods based on our visual inspection (it still has quite high MAPE) with an increased amount of data. The PIs from all four models include the observed numbers of recoveries for all forecast periods. Also, the forecasting accuracy metrics for this application are summarized in Table 1. As we do for previous applications, the point forecasts and prediction intervals for the period V (April 30- May 06, 2020) are provided in S7 Table in S1 File.

## Discussion

We have evaluated four different highly cited time series models for forecasting confirmed cases, number of deaths and number of recoveries of COVID-19 for both the USA and Italy. Our application demonstrated the similarities in performance across countries for key COVID-19 outcomes but also elucidated where predictive performance may be lacking in certain models based on data accrual and sudden, sharp changes in trajectory. Although past publications have largely focused on the forecasting the number of the confirmed cases, we extended our study by considering number of deaths and recoveries. We focused on the data for two countries; however, we illustrated how our approach can be applied to data arising from any country or worldwide data. Similarly, the models considered in our work can be implemented on new data as it becomes available. The forecasting analyses should be updated as the data accumulate periodically to increase accuracy of forecasting, since this is an ongoing pandemic and additional data would improve forecasting performance of these models.

Using multiple models enabled us to test and compare their prediction accuracy and make an optimal selection. Our study showed how selecting a COVID-19 prediction model among existing, powerful options for time series models can readily assist in decision-making through projections regarding important issues such as intervention-induced changes in the spread of disease or making plans for controlling the pandemic and returning to normal life [5]. Such models could be useful in instances where knowledge is limited regarding the progress of the pandemic in a country or the world; assumptions about the future of this emergent disease (COVID-19 pandemic) can be made based on these established models and their available implementations on an open-source platform. Although we considered a breadth of models and outcomes, our work did not account for individualized factors that affect death and recovery. Incorporating such factors is expected to improve accuracy and narrow the width of PIs. Subject-level data applications are needed to address this issue. We should note that these approaches can produce forecasts for longer time periods, such as several weeks or months ahead. We examined forecast durations in a weekly fashion in this work given the nature of the forecasting goal to assess immediate windows of time.

Our study showed that all models resulted in similar overarching conclusions, provided there exists a reasonable amount of historical data available; however, each model exhibited subtle differences. The ARIMA model was the most consistent model across our different applications and could be preferable over the other models if one must pick a single model for application. ARIMA and cubic smoothing spline models had smaller prediction error and narrower PIs, compared to the Holt and TBATS models in the majority of the analyses. This is a surprising finding for the spline models, as this approach tends to perform worse in extrapolation settings. Indeed, these models were not robust in all settings. The TBATS model had quite large widths on the PIs especially when a limited amount of data was available, which is not preferable in applications with constant data accrual. Wider intervals correspond to a higher degree of uncertainty for the point forecasts. On the other hand, the Holt model always had a substantially larger AIC, which indicates poorer model fit, compared to the others for both the USA and Italy applications. TBATS and Spline models mostly resulted in similar AIC values and were often slightly smaller than the AIC of the ARIMA model (the smaller AIC, the better model fit) in the analysis for the USA data. The ARIMA model had the smallest AIC in all forecast periods for the Italy application. However, the model that has better fit (smaller AIC) does not always have a better prediction performance. One should judiciously select a model that has smaller prediction error while better reflecting the nature of the data. The forecasting accuracy metrics of all four models mostly improved with the increased amount of available data as days pass during this pandemic in both the USA and Italy. The unstable patterns in historical data on the number of deaths are more likely to worsen the accuracy of forecasts, but for the future forecasts these models are capable of accounting for major changes in the historical trajectory. In conclusion, these readily available models that can be performed efficiently at any time for any country or worldwide have reasonable accuracy for short-term forecasting of this pandemic, which should not be ignored.

## Supporting information

S1 File (DOCX)
