# Supplementary material for: Forecasting COVID-19 confirmed cases, deaths and recoveries: Revisiting established time series modeling through novel applications for the USA and Italy
Source: PLoS One. 2021 Jan 7;16(1):e0244173. doi: 10.1371/journal.pone.0244173 (PMC7790225; doi:10.1371/journal.pone.0244173)
Supplement: S1 File — (DOCX) [file pone.0244173.s001.docx]

**S1 File**

**Figure S1.** Observed confirmed cases, deaths, and recoveries in the USA and Italy between Jan 22- Apr 29.

**
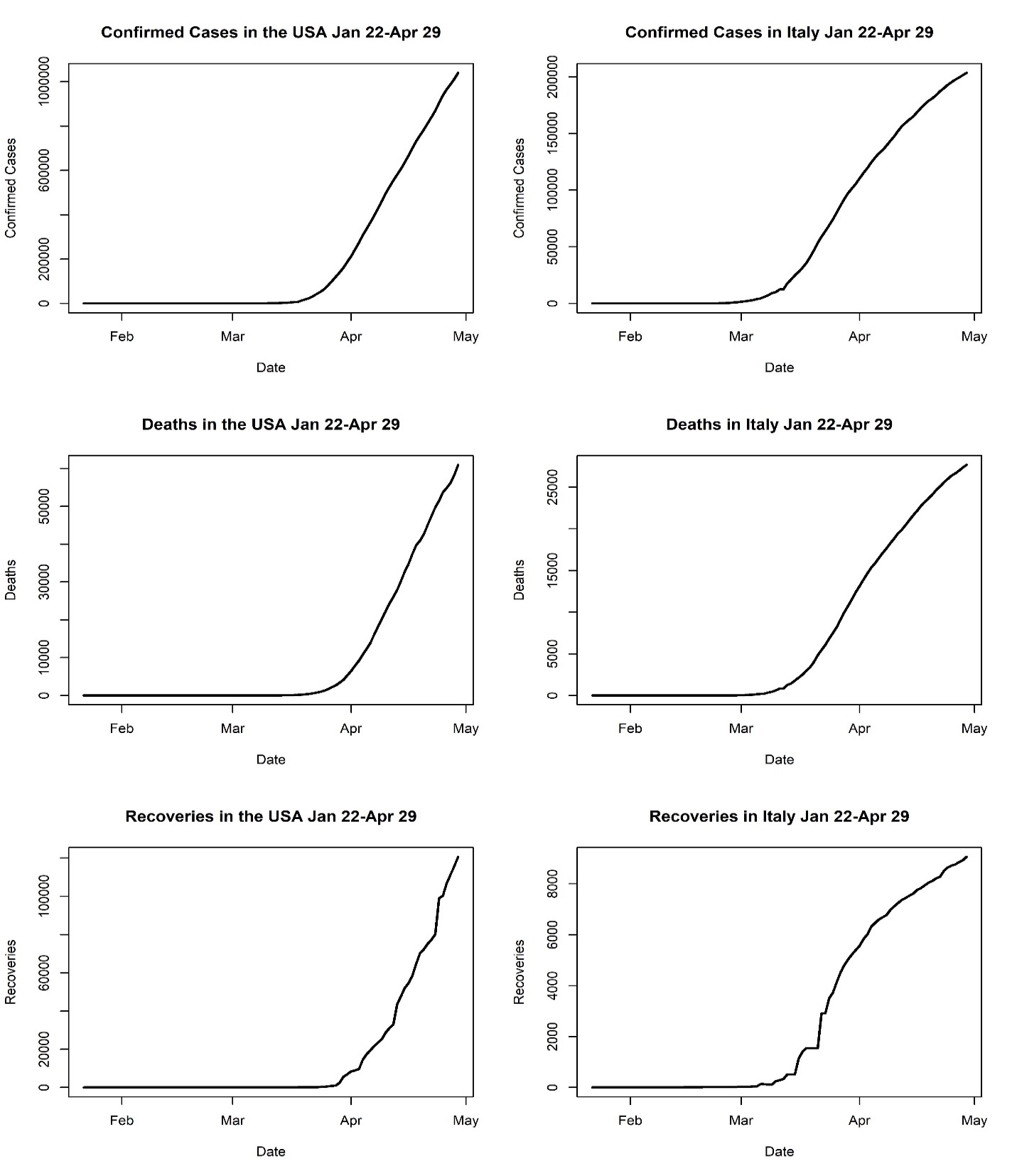
**

**Table S1.** Overview of time series models considered for COVID-19 predictions.

| Time series model | Equation | R implementation |
| --- | --- | --- |
| ARIMA | (1) | auto.arima(df) |
| Holt | (2) | holt(df) |
| Spline | (3) | splinef(df) |
| TBATS | (4) | tbats(df) |

These functions are available in forecast package and *df stands for data frame

**Model Equations:**

**1. ARIMA model**

The response variable that will be explained in time$t$ can modelled with,

| $y_{t}=c+\phi_{1}y_{t-1}+ ... +\phi_{p}y_{t-p}+\theta_{1}e_{t-1}+ ... +\theta_{q}e_{t-q}+e_{t}$ | (1) |
| --- | --- |

where$c$ is intercept; $\phi$ is coefficient of each parameter$p$; 𝜽 is coefficient of each parameter$q$; and$e_{t}$is the residuals or errors in time $t.$ We fit this model by using *auto.arima* function in the forecast package of R which returns the best ARIMA model based on information complexity criteria.

**2. Holt model**

This method involves a forecast equation and two smoothing equations (one for the level and one for the trend);

| $y_{t}=l_{t-1}+b_{t-1}+e_{t},$ | (2) |
| --- | --- |

where$l_{t}=\alpha y_{t}+(1-\alpha)(l_{t-1}+b_{t-1})$ and$b_{t}=\beta^{*}(l_{t}-l_{t-1})(1-\beta^{*}) b_{t-1}$.

Above$l_{t}$denotes an estimate of the level of the series (response variable) at time $t$, $b_{t}$ denotes an estimate of the trend of the series at time$t$,$e_{t}$is the residuals or errors in time $t$, $\alpha$ is the smoothing parameter for the level, 0$\leq$α$\leq1$, and $\beta^{*}$ is the smoothing parameter for the trend 0$\leq\beta^{*}\leq1$. And, the $h$step ahead forecast can be obtained with,$y_{t+h|t}=l_{t}+\mathrm{hb}_{t}.$ This model was fitted by using the *holt* function in the forecast package of R for our applications.

**3. Cubic smoothing Splines**

A cubic smoothing spline for equally spaced times series can be defined as the function $\hat{f}(t)$ which minimizes,

| $\sum_{t=1}^{n} {(y_{t}-f(t))}^{2}+\lambda\int{(f^{''}(u))}^{2}$du | (3) |
| --- | --- |

over all twice-differentiable functions$f$on $S$where [1, n]$\subseteq$ $S$⊆R. In (3), λ is the smoothing parameter which controls the rate of exchange between the residual error described by the sum of squared residuals and local variation represented by the square integral of the second derivative of$f.$ For more details of this model, one can see [9]. The model was fitted by using the *splinef* function in the forecast package of R.

**4. TBATS model**

Under this model, the response variable of interest can be modelled as below,

| $y_{t}^{\lambda}=I_{t-1}+\phi b_{t-1}+\sum_{i=1}^{T} {s_{t-m_{i}}}^{(i)}+d_{t},$ | (4) |
| --- | --- |
| ${I_{t}=I}_{t-1}+\phi b_{t-1}+\alpha d_{t}$, $b_{t}=\phi b_{t-1}+\beta d_{t}$, and $d_{t}=\sum_{i=1}^{p} \psi_{i}d_{t-i}+\sum_{i=1}^{q} \theta_{i}e_{t-i}$+$e_{t}$,  where${s_{t}}^{(i)}=\sum_{j=1}^{k_{i}} {s_{j,t}}^{(i)}$, ${s_{j,t}}^{(i)}={s_{j,t-1}}^{(i)}\cos\left( \omega_{i} \right)+{s_{j,t-1}}^{*(i)}\sin\left( \omega_{i} \right)+{\gamma_{1}}^{(i)}d_{t}$,  ${s_{j,t}}^{*(i)}=-{s_{j,t-1}}^{(i)}\sin\left( \omega_{i} \right)+{s_{j,t-1}}^{*(i)}\cos\left( \omega_{i} \right)+{\gamma_{2}}^{(i)}d_{t}$, and$\omega_{i}=2\pi j/m_{i}$. |  |

Above $y_{t}^{\lambda}$ denotes the time series at moment$t$ (Box-Cox transformed), ${s_{t}}^{(i)}$ denotes ith seasonal component, $I_{t}$ represents local level, $b_{t}$ represents trend with damping, $e_{t}$ is the residuals or errors in time $t$. $T$ is amount of seasonalities, $m_{i}$is length of the i^th^ seasonal period, $k_{i}$is amount of harmonics for the i^th^ seasonal period, λ is a parameter Box-Cox transformation, $\alpha$and β are smoothing parameters, 𝝓 is trend damping, $\theta_{i}$and $\psi_{i}$are ARMA(p, q) coefficients, ${\gamma_{1}}^{(i)}$ and ${\gamma_{2}}^{(i)}$ are the seasonal smoothing parameters. Similar to other model, TBATS model is fitted by using *tbats* function in the forecast package of R.

**The results for the forecasting period V (April 30-May 6)**

**Table S2.** Forecasted confirmed cases and their prediction intervals of the USA application for forecasting period V with all the models.

|  | **ARIMA** | | | **TBATS** | | |
| --- | --- | --- | --- | --- | --- | --- |
| **Days** | **Forecast** | **90 %** LB | **90 %** UB | **Forecast** | **90 %** LB | **90 %** UB |
| Apr 30 | 1067236 | 1063233 | 1071239 | 1065944 | 1052155 | 1079832 |
| May 1 | 1094563 | 1085613 | 1103513 | 1092750 | 1067332 | 1118499 |
| May 2 | 1121890 | 1106914 | 1136866 | 1119924 | 1079880 | 1160774 |
| May 3 | 1149217 | 1127294 | 1171140 | 1147467 | 1090338 | 1206208 |
| May 4 | 1176544 | 1146860 | 1206228 | 1175378 | 1098971 | 1254627 |
| May 5 | 1203871 | 1165689 | 1242053 | 1203661 | 1105946 | 1305956 |
| May 6 | 1231198 | 1183838 | 1278558 | 1232314 | 1111385 | 1360165 |

|  | **Splines** | | | **Holt** | | |
| --- | --- | --- | --- | --- | --- | --- |
| **Days** | **Forecast** | **90 %** LB | **90 %** UB | **Forecast** | **90 %** LB | **90 %** UB |
| Apr 30 | 1067205 | 1063204 | 1071205 | 1067236 | 1063172 | 1071299 |
| May 1 | 1094479 | 1085886 | 1103472 | 1094562 | 1085476 | 1103649 |
| May 2 | 1122154 | 1107378 | 1136929 | 1121889 | 1106686 | 1137093 |
| May 3 | 1149628 | 1127908 | 1171348 | 1149216 | 1126960 | 1171472 |
| May 4 | 1177102 | 1147600 | 1206604 | 1176543 | 1146408 | 1206678 |
| May 5 | 1204577 | 1166539 | 1242614 | 1203870 | 1165108 | 1242632 |
| May 6 | 1232051 | 1184787 | 1279315 | 1231197 | 1183119 | 1279275 |

**Table S3.** Forecasted confirmed cases and their prediction intervals of Italy for forecasting period V with all the models.

|  | **ARIMA** | | | **TBATS** | | |
| --- | --- | --- | --- | --- | --- | --- |
| **Days** | **Forecast** | **90 %** LB | **90 %** UB | **Forecast** | **90 %** LB | **90 %** UB |
| Apr 30 | 205679 | 204336 | 207021 | 205667 | 203918 | 207417 |
| May 1 | 207766 | 205274 | 210258 | 207747 | 204677 | 210823 |
| May 2 | 209854 | 205867 | 213841 | 209830 | 205235 | 214441 |
| May 3 | 211941 | 206284 | 217598 | 211917 | 205610 | 218251 |
| May 4 | 214029 | 206503 | 221554 | 214006 | 205820 | 222238 |
| May 5 | 216116 | 206558 | 225674 | 216098 | 205878 | 226390 |
| May 6 | 218204 | 206457 | 229951 | 218194 | 205793 | 230698 |

|  | **Splines** | | | **Holt** | | |
| --- | --- | --- | --- | --- | --- | --- |
| **Days** | **Forecast** | **90 %** LB | **90 %** UB | **Forecast** | **90 %** LB | **90 %** UB |
| Apr 30 | 205662 | 204312 | 207011 | 205660 | 204293 | 207026 |
| May 1 | 207746 | 205092 | 210400 | 207730 | 205208 | 210252 |
| May 2 | 209831 | 205539 | 214123 | 209801 | 205943 | 213659 |
| May 3 | 211915 | 205725 | 218106 | 211871 | 206511 | 217231 |
| May 4 | 214000 | 205687 | 222313 | 213942 | 206930 | 220954 |
| May 5 | 216084 | 205448 | 226721 | 216012 | 207209 | 224815 |
| May 6 | 218169 | 205024 | 231314 | 218083 | 207360 | 228806 |

**Table S4.** Forecasted number of deaths and their prediction intervals of the USA for forecasting period V with all the models.

|  | **ARIMA** | | | **TBATS** | | |
| --- | --- | --- | --- | --- | --- | --- |
| **Days** | **Forecast** | **90 %** LB | **90 %** UB | **Forecast** | **90 %** LB | **90 %** UB |
| Apr 30 | 63215 | 62712 | 63717 | 63301 | 62800 | 63801 |
| May 1 | 65387 | 64360 | 66413 | 65490 | 64480 | 66499 |
| May 2 | 67694 | 66154 | 69234 | 67679 | 66161 | 69197 |
| May 3 | 69997 | 67869 | 72124 | 69868 | 67808 | 71927 |
| May 4 | 72256 | 69458 | 75055 | 72056 | 69419 | 74469 |
| May 5 | 74527 | 71004 | 78049 | 74245 | 70991 | 77500 |
| May 6 | 76908 | 72513 | 81104 | 76434 | 72526 | 80342 |

|  | **Splines** | | | **Holt** | | |
| --- | --- | --- | --- | --- | --- | --- |
| **Days** | **Forecast** | **90 %** LB | **90 %** UB | **Forecast** | **90 %** LB | **90 %** UB |
| Apr 30 | 63521 | 62997 | 64046 | 63305 | 62785 | 63826 |
| May 1 | 66134 | 65056 | 67212 | 65644 | 64647 | 66641 |
| May 2 | 68747 | 66979 | 70518 | 67983 | 66438 | 69528 |
| May 3 | 71360 | 68785 | 73935 | 70322 | 68162 | 72482 |
| May 4 | 73960 | 70499 | 77447 | 72661 | 69823 | 75499 |
| May 5 | 76586 | 72126 | 81045 | 75000 | 71428 | 78572 |
| May 6 | 79199 | 73675 | 84722 | 77339 | 72979 | 81699 |

**Table S5.** Forecasted number of deaths and their prediction intervals of Italy for forecasting period V with all the models.

|  | **ARIMA** | | | **TBATS** | | |
| --- | --- | --- | --- | --- | --- | --- |
| **Days** | **Forecast** | **90 %** LB | **90 %** UB | **Forecast** | **90 %** LB | **90 %** UB |
| Apr 30 | 27994 | 27851 | 28136 | 28008 | 27683 | 28335 |
| May 1 | 28293 | 28060 | 28526 | 28343 | 27872 | 28818 |
| May 2 | 28586 | 28246 | 28927 | 28680 | 27997 | 29368 |
| May 3 | 28874 | 28405 | 29344 | 29018 | 28078 | 29969 |
| May 4 | 29158 | 28532 | 29785 | 29358 | 28124 | 30612 |
| May 5 | 29439 | 28624 | 30255 | 29699 | 28140 | 31290 |
| May 6 | 29718 | 28680 | 30757 | 30042 | 28128 | 32003 |

|  | **Splines** | | | **Holt** | | |
| --- | --- | --- | --- | --- | --- | --- |
| **Days** | **Forecast** | **90 %** LB | **90 %** UB | **Forecast** | **90 %** LB | **90 %** UB |
| Apr 30 | 28026 | 27878 | 28173 | 28029 | 27879 | 28180 |
| May 1 | 28367 | 28116 | 28619 | 28376 | 28119 | 28633 |
| May 2 | 28709 | 28327 | 29092 | 28723 | 28322 | 29124 |
| May 3 | 29051 | 28516 | 29586 | 29070 | 28498 | 29641 |
| May 4 | 29393 | 28688 | 30097 | 29417 | 28653 | 30180 |
| May 5 | 29734 | 28844 | 30625 | 29764 | 28789 | 30738 |
| May 6 | 30076 | 28985 | 31167 | 30110 | 28907 | 31313 |

**Table S6.** Forecasted number of recoveries and their prediction intervals of the USA for forecasting period V with all the models.

|  | **ARIMA** | | | **TBATS** | | |
| --- | --- | --- | --- | --- | --- | --- |
| **Days** | **Forecast** | **90 %** LB | **90 %** UB | **Forecast** | **90 %** LB | **90 %** UB |
| Apr 30 | 126108 | 122175 | 130041 | 127408 | 110934 | 145770 |
| May 1 | 131322 | 125858 | 136787 | 134504 | 108041 | 165881 |
| May 2 | 136537 | 129481 | 143592 | 141911 | 105960 | 186952 |
| May 3 | 141751 | 133025 | 150476 | 149640 | 104018 | 209908 |
| May 4 | 146965 | 136485 | 157445 | 157699 | 102012 | 235234 |
| May 5 | 152179 | 139860 | 164498 | 166099 | 99861 | 263310 |
| May 6 | 157393 | 143152 | 171634 | 174851 | 97535 | 294496 |

|  | **Splines** | | | **Holt** | | |
| --- | --- | --- | --- | --- | --- | --- |
| **Days** | **Forecast** | **90 %** LB | **90 %** UB | **Forecast** | **90 %** LB | **90 %** UB |
| Apr 30 | 125205 | 120926 | 129485 | 126130 | 122198 | 130062 |
| May 1 | 129699 | 122691 | 136707 | 131348 | 125943 | 136754 |
| May 2 | 134192 | 123713 | 144672 | 136566 | 129625 | 143508 |
| May 3 | 138686 | 124177 | 153195 | 141785 | 133230 | 150340 |
| May 4 | 143180 | 124174 | 162185 | 147003 | 136752 | 157253 |
| May 5 | 147673 | 123757 | 171590 | 152221 | 140194 | 164249 |
| May 6 | 152167 | 122962 | 181372 | 157439 | 143554 | 171324 |

**Table S7.** Forecasted number of recoveries and their prediction intervals of Italy for forecasting period V with all the models.

|  | **ARIMA** | | | **TBATS** | | |
| --- | --- | --- | --- | --- | --- | --- |
| **Days** | **Forecast** | **90 %** LB | **90 %** UB | **Forecast** | **90 %** LB | **90 %** UB |
| Apr 30 | 9147 | 8833 | 9460 | 9152 | 8702 | 9606 |
| May 1 | 9248 | 8809 | 9687 | 9252 | 8623 | 9890 |
| May 2 | 9346 | 8761 | 9930 | 9353 | 8539 | 10181 |
| May 3 | 9445 | 8712 | 10177 | 9454 | 8448 | 10482 |
| May 4 | 9543 | 8654 | 10432 | 9555 | 8347 | 10794 |
| May 5 | 9642 | 8589 | 10694 | 9656 | 8237 | 11118 |
| May 6 | 9740 | 8516 | 10964 | 9758 | 8119 | 11453 |

|  | **Splines** | | | **Holt** | | |
| --- | --- | --- | --- | --- | --- | --- |
| **Days** | **Forecast** | **90 %** LB | **90 %** UB | **Forecast** | **90 %** LB | **90 %** UB |
| Apr 30 | 9160 | 8810 | 9509 | 9147 | 8833 | 9461 |
| May 1 | 9273 | 8654 | 9892 | 9245 | 8809 | 9680 |
| May 2 | 9386 | 8426 | 10345 | 9342 | 8776 | 9908 |
| May 3 | 9498 | 8144 | 10853 | 9440 | 8734 | 10145 |
| May 4 | 9611 | 7816 | 11407 | 9537 | 8683 | 10391 |
| May 5 | 9724 | 7447 | 12001 | 9635 | 8624 | 10645 |
| May 6 | 9837 | 7041 | 12634 | 9732 | 8557 | 10908 |

**Supplemental R code**

The R script below is based on the analysis of the data downloaded from <https://github.com/CSSEGISandData/COVID-19>. Variables that we have used from this dataset are defined in the manuscript.

# install packages

library(forecast)

#load data set and define subset datasets for each forecasting period

df<-~ time_series_covid19_confirmed_global.csv

#Below *h*: is forecasting window and *level*: the level of prediction interval desired to obtain

# Automatic ARIMA forecasts

model1<-auto.arima(df)

forecast_aARIMA<- forecast(model1, h=7, level = 90)

accuracy(forecast_aARIMA)

#TBATS forecasts

model2<-tbats(df)

forecast_TBATS<- forecast(model2, h=7, level = 90)

accuracy(forecast_TBATS)

#forecasting with Holt model

model3<- holt(df)

forecast_holt <- holt(model3, h=7, level = 90)

accuracy(forecast_holt)

# Cubic smoothing spline model

forecast_CSsplines<- splinef(df, h=7, level = 90)

accuracy(forecast_CSsplines)
